# Permissive nicotine regulation as a complement to traditional tobacco control

**DOI:** 10.1186/1471-2458-5-18

**Published:** 2005-02-24

**Authors:** Walton Sumner

**Affiliations:** 1 Department of Medicine, Washington University School of Medicine, St. Louis, Missouri, USA

## Abstract

**Background:**

Cigarette smoking takes a staggering toll on human health and attracts considerable public health attention, yet real solutions seem distant. The 2004 Family Smoking Prevention and Tobacco Control Act (US Senate bill S2461) would have given the US Food and Drug Administration limited authority to regulate cigarettes to "protect the public health." However, such legislation is unlikely to substantially reduce smoking or related deaths.

**Discussion:**

The past 500 years of tobacco control efforts demonstrate that nicotine prohibition is a practical impossibility for numerous reasons, state revenue being one of the most ominous. The FDA already has regulatory authority over pharmaceutical grade nicotine products, and requires pharmacists to dispense the most addictive of these only with prescriptions. Meanwhile, every corner store can sell far more addictive and dangerous cigarettes to any adult. The FDA could immediately increase competition between cigarettes and clean nicotine products by approving available nicotine products for over-the-counter sales to adults. Similarly permissive regulation of cigarettes and addictive nicotine products will reduce tobacco use and improve smokers' health, but increase nicotine use in the population. Fortunately, restricted youth access and accurate labeling of nicotine's absolute risks will dissuade many non-smokers from experimenting with it, while accurate depiction of its risks relative to cigarette smoking will encourage many smokers to switch. The FDA could take a series of small steps that might ultimately replace a large proportion of cigarette smoking with equally addictive nicotine products, without risking serious public health setbacks. Vaccine, methadone, and injury prevention policies establish relevant public health precedents.

**Summary:**

Cigarettes, or an equally addictive alternative, will be a permanent and common product in most societies. Regulations restricting only the safest addictive nicotine products are hard to justify. Addictive nicotine compliments other tobacco control strategies. Modern tobacco control policies are applicable to addictive nicotine. Controlled trials and test market studies are urgently needed to evaluate addictive nicotine as an alternative to smoking. Meanwhile, legislators should preserve the Food and Drug Administration's option to permit non-prescription sales of addictive nicotine.

## Background

Cigarette smoking is a source of worldwide misery [1,2] and revenue for corporations and governments. Landmark tobacco regulatory efforts in the United States include the 1992 Synar Amendment, requiring States to establish and enforce prohibitions on the sale and distribution of tobacco products to persons under 18 years of age [3]. The 1998 Master Settlement Agreement (MSA) prohibited numerous marketing practices, especially those likely to entice youth [4]. The six corporate signatories also promised an endless series of payments to the settling States, currently $8 billion annually. The United States has endorsed the World Health Organizations' 2003 Framework Convention on Tobacco Control (FCTC) [5]. The FCTC enumerates well-studied, politically safe measures such as taxation; limiting youth access; regulating the content, packaging, advertising, and sales of tobacco products; and educating the public about risks. Fire-safe cigarettes [6] are now required in New York. Several states and many municipalities now require smoke-free workplaces.

The recently defeated [7] 2004 Family Smoking Prevention and Tobacco Control Act, S2461 [8], would have established Food and Drug Administration (FDA) regulation of cigarettes for the first time. Although average citizens could easily have taken "FDA regulation" to suggest the extensive power that the FDA holds over pharmaceutical products[9], tobacco regulations would have been weaker. The defeated Act sought publication of brand-specific ingredient information; forbade adding children's favorite flavorings to cigarettes, further restricted teen sales and advertising; and allowed the FDA to regulate cigarettes' nicotine delivery. The FDA would not have been able to ban or eliminate nicotine from cigarettes, nor could the FDA ban a class of tobacco products. Although modestly effective at preventing and ending tobacco use, such measures will not achieve the Healthy People 2010 (HP2010) goal of 12% adult smoking prevalence [10] on schedule [11], if ever. While cigarettes should never again afflict more than a quarter of the United States population, tobacco regulation will abate the current level of carnage only very slowly.

A complimentary strategy called "harm reduction" proposes that smokers could improve their health by frequently substituting less hazardous tobacco products, such as 'smokeless cigarettes,' chewing tobacco, and newer smokeless tobacco products for cigarettes [12-14]. 'Smokeless cigarettes' are neither smokeless nor cigarettes, but complicated devices that release a mixture of chemicals from a heated tobacco substrate and can emit more carbon monoxide than a cigarette [15-18]. For individuals who are determined to inhale nicotine, these devices are probably no worse than cigarettes. However, for individuals who would have quit instead of switching to a smokeless cigarette, the health cost may be large. The Institute of Medicine has called for extensive research to quantify harms and patterns of use of these "potential reduced exposure products" (PREP) [19]. In contrast, chewing tobacco is clearly safer than smoking cigarettes [20,21], exposing users to as little as 2% of the risks of smoking [22]. The newest smokeless tobacco products are discrete enough to use in almost any social setting. Smokeless tobacco products are much less popular than smoking in the United States for a variety of reasons, including restrictions on advertising. However, the most important limitation of smokeless tobacco may be absorption through the nicotine through systemic veins rather than the pulmonary vasculature. All current pharmaceutical nicotine products share this limitation.

Some tobacco control advocates suggest a third strategy: national policies encouraging competition between safer nicotine products and cigarettes [20,23-34]. Typically, this involves a "level playing field" with similar regulations for pharmaceutical grade nicotine delivery systems and cigarettes. Advocates cite evidence that health risk correlates with exposure to tobacco's combustion and curing by-products [35-38], with nicotine replacement products posing the least risk [39,40].

There are different ways to level this playing field. At one extreme, the FDA could increase regulation of cigarettes to match that of nicotine products. This is politically challenging, as the FDA discovered in the 1990's [41], The Family Smoking Prevention and Tobacco Control Act divided both the tobacco industry and the public health community. Altria, corporate parent of Philip Morris, was the only tobacco company to endorse this legislation. Calling a similar bill the "Marlboro monopoly act" [42], critics suspect that Altria hoped to discourage competition, for instance by encumbering smaller producers with regulations, by prohibiting relative safety claims sought by chewing tobacco manufacturers [43], or by discouraging new products with stringent new standards [44,45]. Other critics believe that the Act safeguarded certain cigarette marketing and legal defense strategies [9], or paved the way for the company's unproven 'smokeless cigarettes,' potentially ushering in another generation of suffering.

At another extreme, the FDA could regulate other nicotine products more permissively, as cigarettes have been regulated. The FDA has that authority, but would face criticism for facilitating a common addiction. For instance, the FDA could approve non-prescription nicotine nasal sprays and slow acting inhalers for indefinite use by adults. These nicotine replacement therapies have excellent safety records and low potential for addicting non-smokers, although a few ex-smokers become addicted. The widespread misconception that nicotine prohibition is practical and desirable makes it politically difficult for the FDA to pursue this path.

## Discussion

### Problems with nicotine prohibition

A recent Lancet editorial called for the criminalization of tobacco products [46]. However, a litany of centuries-old problems with prohibition provides a strong argument for permissive nicotine regulation as an alternative or essential prerequisite to prohibition. Many people will pay high prices to a coalition of suppliers and governments to obtain tobacco cigarettes that efficiently deliver nicotine, a drug perceived to provide some benefits, in spite of substantial immediate and long-term risks of smoking. While comprehensive public smoking restrictions are possible, and some companies may exit the tobacco trade, traditional tobacco control and litigation may not provide additional large public health gains in the United States, and prohibition is a practical impossibility.

The first problem is that historically, smokers accept personal and public hazards that make the dangers we associate with tobacco look quaint. After Christopher Columbus failed to control his crew's tobacco use, monarchs from England to China tried to contain the weed by execution, disfigurement, exile, and onerous taxation [47]. Tobacco trade drained monarchs' wealth, compromising national security, and smoking accidents incinerated whole cities. Tobacco use spread anyway.

The second problem is that risk-tolerant smokers are no small group. Smoking persists where Mormon and Islamic prohibitions discourage it [48,49]. If 70% of smokers in the USA want to quit [50]. then 30% of smokers, more than 6% of the adult population, do not want to quit.

The third problem is a common genetic predisposition to nicotine addiction [51]. The modifiable risk factors we associate with smoking initiation and persistence – parent behavior, peer pressure, role models, advertising, accessibility, repeated exposure, and perceived norms – were irrelevant to Christopher Columbus' crew and the first tobacco users across Eurasia. Risk factor modification will not alter genetically predisposed users' fascination with their first tobacco products.

The fourth problem is that so much money is involved. When state and local taxes raised New York City cigarette prices to US$7 per pack, tax revenues and black marketing both increased [52]. During the invasion of Iraq, stressed US soldiers reportedly paid up to US$50 per pack [53]. The black market for tobacco in Colorado prisons may achieve a 45,000% markup [54]. This industry makes large profits selling a simple product. If liability claims bankrupt corrupt companies, new suppliers will fill the void and try to avoid predecessor's mistakes. Black markets will undermine the benefits of high taxation or prohibition.

The fifth problem is that government taxation compromises tobacco control efforts. Historically, States willingly trade citizens' health for wealth. Seventeenth century monarchs who opposed smoking relented as tobacco tax revenue accumulated. The States demonstrated the same perverse values with petitions to protect their MSA payments when an Illinois court threatened Altria with a US$12 billion bond [55]. The States will predictably protect the MSA corporate signatories from new competitors, for instance with taxes targeting generic brands. Tax revenues diminish prospects for even slowing the growth of tobacco sales in Africa, Eurasia, and South America, and prohibition in those areas is currently impossible.

The sixth problem is the lack of a popular mandate for prohibition. Tobacco control advocates in the United States are rightfully pleased with smoking's declining prevalence, reduced teen smoking, rising cigarette prices, spreading restrictions on public smoking, and fire-safe cigarette initiatives. However, none of this demonstrates popular support for prohibition. Citizens will balk at limiting the supposedly personal choice to smoke in private. Midwesterners have already rejected modest cigarette tax hikes [56]. Furthermore, most taxes and MSA payments subsidize programs that benefit non-smokers [57]. Prohibition would eliminate that subsidy and require non-smokers to pay for enforcement, a very unlikely prospect.

The seventh problem is that nicotine use could have a favorable risk-benefit profile for some informed users. Nicotine causes a mild euphoria without intoxication, in contrast to more tightly regulated drugs: it does not destroy relationships as intoxicating drugs routinely do. Many smokers may use nicotine to treat various problems [58-60] including depression [61], attention deficits [62], other mental illness [63], symptomatic systemic diseases [64], or to control weight [65]. Nicotine users may perform some tasks better, especially those involving vigilance and rapid visual cue processing [66,67]. There is uncertainty regarding many of these benefits [68]. Nevertheless, expected benefits are politically hard to withhold. If some of these benefits are real, nicotine prohibition may not even be desirable.

Nicotine accounts for very few of the long-term hazards of smoking. A smokeless tobacco proponent has likened nicotine's risks to the risks of consuming caffeine [54]. Fetal exposure causes placental constriction and reduced birth size, alters brain development in disturbing ways, and may increase susceptibility to later nicotine addiction [69-76]. Nicotine might contribute to sudden infant death syndrome [69,77-79], destruction of connective tissue [80], modulation of immune function [81], prevention of apoptosis [82,83], and alcohol or other substance abuse [84-86]. Fatal nicotine poisoning is quite unlikely [87-89]. Given that smokeless tobacco users experience only about 2% of the risks of smoking [22], and that inhaled nicotine is similarly benign in animal models [90-92], it is very unlikely that inhaled nicotine could account for even one-tenth of the harms of smoking.

In summary, however desirable tobacco prohibition may be, it is hopelessly impractical – unless smokers, governments, and producers have an equally satisfying alternative.

### Stifling innovation

Legal and regulatory pressures have prevented the development of meaningful alternatives to cigarettes. Directed to approve drugs as "safe and effective" for specific indications, and to ignore tobacco, the FDA has had little reason to approve chronic, addictive nicotine. Although the FDA approves more dangerous drugs for specified indications, without an indication there is no benefit to weigh against any nicotine risk. Consequently the FDA requires prescriptions for slow acting inhalers and nasal spray because of a small risk of inconsequential addiction in ex-smokers. Unfortunately, seemingly risk averse regulation of nicotine forces public health policies to rely on difficult smoking prevention, cessation, use restrictions, and treatment. The net effect is a risky public health policy, and very slow development of new nicotine delivery systems [32]. Another unfortunate side effect has been that companies put tobacco into any device that they do not want regulated, particularly, 'smokeless cigarettes,' even if the device would be safer without tobacco. In permissive nicotine regulation, the FDA's missing indication is to improve nicotine addicts' safety. As prescription methadone substitutes for illegal, immediately incapacitating heroin, non-prescription nicotine could substitute for legal, slowly injurious cigarettes.

Tort threats have also delayed product improvement efforts within the industry. In 1963 an industry lawyer, anticipating condemnation in the 1964 Surgeon General's report, suggested competing on safe nicotine delivery, but was overruled [93]. Product liability and regulatory issues pushed tobacco companies into pointless projects including filters, light cigarettes, "smokeless cigarettes", nicotine-free cigarettes, and fabricating a controversy over the health risks of smoking.

### Haddon matrix

In the mid 1960's, motor vehicle accidents were also taking a terrific health toll in the United States. While alternatives to driving existed, no one expected private automobiles to disappear. Instead, systematic efforts transformed traffic safety. Haddon's matrix illustrated how complimentary strategies could work together to reduce the morbidity and mortality of driving [94]. Table 1 shows a matrix with two axes representing time and objects. Most injury control efforts fall into one of the nine cells, although some cells are empty. Before an accident, typical preventive efforts remove or modify unsafe drivers, vehicles, and road conditions. During an accident a vehicle's design and environmental safeguards may prevent or limit injuries. After an accident, prompt medical attention limits the morbidity and mortality of the injuries that still occur.

**Table 1:** Haddon's Matrix for Reducing Traffic Accident Injuries

	**Driver/Passenger**	**Vehicle**	**Environment**
**Before Accident**	Licensing Stops, tickets, arrests Drug, alcohol screening Physician advice	Running lights Antilock brakes High traction tires Vehicle inspections Impact avoidance	Road design Road maintenance Road lighting, marking Traffic law Police patrols
**During Accident**	----	Bumpers Seat belts Airbags Crumple zones Fuel containment	Deformable barriers Fences
**After Accident**	----	Fuel containment	Telecommunications Emergency stabilization Emergency transport Trauma centers

For tobacco control, smoking replaces accidents as the focal event (Table 2 – anticipated strategies are *italicized*) [95]. As with accidents, cells contain only partially effective interventions. Many people experiment with tobacco in spite of efforts to prevent tobacco use. During smoking, smokers quit infrequently in spite of warnings and medications. More effective smoking cessation products may appear [96,97]. but some nicotine use will persist. In the center cell, hazardous cigarettes easily dominate all available nicotine replacement products [98]. Available and anticipated harm reduction products may be safer, but addictive nicotine would be safer still. After injury, detection of disease may be slow, and treatments for tobacco-related diseases are seldom curative. The matrix highlights the problems of incomplete prevention, unsafe nicotine sources, and poor treatment options, and demonstrates why permissive nicotine regulation could benefit public health: when prevention fails, results are bad. Making nicotine addiction as safe as possible would make prevention failures less disastrous.

**Table 2:** Haddon's matrix adapted to tobacco control

	**Potential user**	**Nicotine delivery device**	**Environment**
**Before Addiction**	Addiction education Disease education Counter-advertising	Warning labels Labeling regulations	Youth sales restrictions Taxation Black market policing *Prohibition*
**During Use**	Disease education Cessation advertising Bupropion Nortiptylline *Varenicline *[105], *Rimonabant* [97]	Filters Low tar cigarettes Smokeless tobacco Smokeless cigarettes *Content regulation* Nicotine replacement *Addictive inhalers*	Taxation *Prohibition*
**After Injury**	Disease awareness Cessation advice	Nicotine replacement *Addictive inhalers*	Disease screening Disease treatment *Cures*

### Proposal

For the reasons outlined above, the health benefits of addictive pharmaceutical grade nicotine products would likely outweigh the harms. If so, the FDA could improve public health by regulating nicotine much as the government has regulated cigarettes.

A permissive nicotine regulatory policy would allow sales of pharmaceutical grade nicotine delivery systems to adults without a prescription. The FDA could suggest warnings appropriate for classes of products and delivery system constituents. Manufacturers would accurately label contents, and would be legally liable for undisclosed harms caused by the delivery system and constituents other than nicotine, as with any pharmaceutical product. The FDA could prohibit inherently risky delivery systems, and would undertake a full, traditional review of nicotine systems that give users a faster or higher peak arterial level of nicotine than cigarettes. The FDA would also review additives intended to provide antidepressant or other effects familiar to smokers [99,100]. Youth marketing and access would be illegal, as with cigarettes. The Drug Enforcement Agency would have no jurisdiction over non-intoxicating nicotine products, just as it has no interest in tobacco. Most public and workplace restrictions on cigarette smoking would be irrelevant to other nicotine delivery systems.

States could tax cigarettes and nicotine at different rates, in theory recovering expenses related to each product. This tax policy would discourage cigarette use, encourage switching to nicotine, and maintain some State revenue as cigarette sales decline. A satisfying, safe, legal, and affordable alternative to cigarettes would discourage black markets. The FDA and Federal Trade Commission could permit advertising of nicotine as an alternative to smoking, and monitor relative harm claims. Nicotine manufacturers would pay for monitoring of adverse health effects from their products until remaining health questions are answered.

The FDA could implement a permissive policy in a series of simple, informative steps. First, the FDA could immediately approve non-prescription sales of the existing nicotine spray and inhaler, and similar competitors, with restraints consistent with the Synar amendment, the MSA, and the FCTC. In particular, it should require disclosure of known nicotine risks, such as addiction, invite comparisons to smoking risks, and forbid marketing to minors.

Data collected during this first step will inform subsequent steps. Economists have shown that the price of nicotine gum and patches affects demand for cigarettes [101]. This means that some smokers will substitute less expensive but very slow acting nicotine replacement products for cigarettes. Non-prescription access to more addictive and competitively priced nicotine should increase this substitution, especially if addicts can purchase a one day supply of about 20 mg. Clinical trials and post-marketing studies of non-prescription use of the spray and inhaler would begin to answer questions about nicotine and pregnancy, substance abuse, and heart disease. Surveillance studies would determine whether clean nicotine options lead more people to smoke, to smoke longer, or to use nicotine during pregnancy. While the Institute of Medicine recommends years or decades of study to quantify the risks of "smokeless cigarettes", the most important remaining uncertainties about nicotine might be answered in a few years. Data collected during this first step should document the relative safety of addiction to pharmaceutical nicotine versus cigarettes, the market share of each product, and the increase in total nicotine use. Analyses using these data will predict the consequences of more permissive regulation [102].

If experience with the current nicotine spray and inhaler is reassuring, the FDA can take a second step, to approve clearly addictive nicotine inhalers for non-prescription use. These fast acting inhalers will deliver nicotine in a powder [103] or aerosol to the alveoli, as pulmonary inhalers deliver steroids and beta agonists. Fast acting nicotine inhalers would be subject to the same marketing requirements and surveillance described above.

Several trends could develop that encourage smokers to switch to addictive nicotine inhalers, while limiting recruitment of non-smokers. Smokers will appreciate the healthier alternative to cigarettes, especially as they develop smoking related illnesses, while warning labels will discourage casual experimentation by non-smokers. Smoke-free workplace regulations would spread with less controversy, as fast acting inhalers give smokers a reasonable alternative. Employers might voluntarily forbid smoking to reduce health care and workman's compensation costs; reduce workplace fires; and increase productivity by eliminating both smoking breaks and withdrawal symptoms. Physicians might strongly encourage smokers to switch for their own health, primarily, but also for the health of family members. The government might permit more aggressive advertising of nicotine inhalers to further undermine cigarette smoking. Governments will probably find nicotine taxation irresistible, especially if their tobacco revenue declines. Nicotine taxation will raise the cost of experimenting with inhalers beyond some teenager's means. Communities might press smokers to switch so that fire departments, health care providers, and research funding agencies could shift resources to many other pressing problems. Ultimately, palatable inhaled nicotine products could finally allow governments to ban tobacco cigarettes.

The public health benefits of these policy shifts could be substantial. We can estimate the long term relative public health burdens of different policies as the product of the risks posed by a delivery system, relative to cigarettes, and the fraction of the adult population using it. Using this formula, the current burden is more than 20% use × 100% risk = 0.2. Healthy People 2010 calls for 12% use × 100% risk = 0.12, about half of the current burden. If nicotine accounted for a surprising 10% of smoking risks, eliminating smoking by quintupling the prevalence of inhaled nicotine use – universal addiction – would cause 100% use × 10% risk = 0.1, a slight improvement over HP2010. A slightly less pessimistic scenario would be that all past and present smokers become inhaled nicotine addicts, but their risk is only twice that of smokeless tobacco users. This scenario is no more than 70% use × 4% risk = 0.028, about a quarter of the HP2010 burden. The most likely scenario is that nicotine risks are about 2% of smoking risks, and that policy changes will slowly shift the probabilities that smokers will attempt and succeed in quitting, that smokers will switch to nicotine indefinitely or use both nicotine and cigarettes, that ex-smokers relapse to nicotine use, and that non-smokers begin using nicotine. If the risks of inhaled nicotine are this low and if at least one smoker can switch to addictive inhaled nicotine for every 50 ex-smokers and non-smokers who start using nicotine, then public health will benefit from permissive nicotine regulation. One public health risk is that more people might begin or continue smoking cigarettes in the belief that inhaled nicotine will provide an easy escape path, but suffer irreparable harm before switching. We should quantify these probabilities through surveillance of the public's behavior and health consequences following each step toward more permissive nicotine regulation.

Although S2461 was defeated, the continuing carnage will motivate similar legislative proposals. Tobacco policies must preserve the possibility of permissive nicotine regulation. Bad legislation could solidify the position of cigarettes, delay safer products, prohibit disclosure of relative harms, or otherwise interfere with market forces that ought to benefit nicotine addicts [104]. The FDA must retain the option to unleash real competition against a disastrous status quo in the tobacco industry.

### Summary

Nicotine use will remain common indefinitely. Pharmaceutical grade nicotine is the safest known substance that could replace cigarettes, but inherently addictive products are required to compliment traditional tobacco control policies effectively. The FDA could apply restrictions, similar to those on cigarettes, to an increasingly addictive portfolio of nicotine products, requiring honest portrayals of absolute and relative risks, and expect a significant reduction in cigarette smoking and related illness with modest recruitment of new nicotine addicts at each step. A brief and focused research effort could solidify the already substantial justification for such permissive nicotine regulation. Meanwhile, federal legislation must not obstruct free market ideals of competitive innovation and informed consumption.

## Competing interests

The author(s) declares that he has no competing interests.

## Pre-publication history

The pre-publication history for this paper can be accessed here:


